# A novel test for assessment of anterolateral rotatory instability of the knee: the tibial internal rotation test (TIR test)

**DOI:** 10.1186/s40634-018-0141-9

**Published:** 2018-08-09

**Authors:** Malou E. Slichter, Nienke Wolterbeek, K. Gie Auw Yang, Jacco A. C. Zijl, Tom M. Piscaer

**Affiliations:** 1 Department of Orthopaedic Surgery, St. Antonius hospital, P.O. Box 2500, 3430 EM Nieuwegein, The Netherlands; 2 Department of Orthopaedic Surgery, Erasmus Medical Center, P.O. Box 2040, 3000 CA Rotterdam, the Netherlands

**Keywords:** Anterolateral rotatory instability, Rotatory laxity, Tibial internal rotation, Anterior cruciate ligament, Anterolateral ligament, Physical examination, Pivot shift, Knee, Ligament, Knee instability

## Abstract

**Background:**

Rotational instability of the knee may persist after anterior cruciate ligament (ACL) reconstruction, which may be due to insufficiency of anterolateral stabilizing structures. However, no reliable diagnostic tool or physical examination test is available for identifying patients with anterolateral rotatory instability (ALRI). As shown in cadaveric studies, static internal rotation of the knee is increased in higher flexion angles of the knee after severing the anterolateral structures. This might also be the case in patients with an ACL-deficient knee and concomitant damage to the anterolateral structures. The objective of this study is to assess anterolateral rotatory instability of the knee during physical examination with a tibial internal rotation test.

**Methods:**

ACL-injured knees of 52 patients were examined by two examiners and side-to-side differences were compared. Both lower legs were internally rotated by applying manual internal rotation torque to both feet in prone position with the knees in 30°, 60° and 90° of flexion. For quantification of the amount of rotation in degrees, a torque adapter on a booth was used. Intra-rater, inter-rater and rater-device agreement were determined by calculating kappa (κ) for the tibial internal rotation test.

**Results:**

Tibial internal rotation is increased in 19.2% of the patients with ACL injury according to the tibial internal rotation test. Good intra-rater agreement was found for the tibial internal rotation test, κ_C_ = 0.63 (95%CI -0.02-1.28), *p* = 0.015. Fair inter-rater agreement was found, κ_F_ = 0.29 (95%CI 0.02–0.57), *p* = 0.038. Good rater-device agreement was found, κ_C_ = 0.62 (95%CI 0.15–1.10), *p* = 0.001.

**Conclusion:**

The tibial internal rotation test shows increased tibial internal rotation in a small amount of patients with ACL injury. Even though no gold standard for assessment of increased tibial internal rotation of the knee is available yet, the test can be of additional value. It can be used for assessment of internal rotatory laxity of the knee as part of ALRI in addition to the pivot shift test. No clinical implications should yet be based on this test alone.

**Electronic supplementary material:**

The online version of this article (10.1186/s40634-018-0141-9) contains supplementary material, which is available to authorized users.

## Background

Significant anterolateral rotational instability (ALRI) may persist after anterior cruciate ligament (ACL) reconstruction with a positive pivot shift test in up to 25% of the patients (Sonnery-Cottet et al., 2015). This might cause functional disability and patient dissatisfaction, and is associated with a reduced rate of return to sport, and an increase in re-injury (Ayeni et al., 2012; Jonsson et al., 2004; Kaplan et al., 1990; Kocher et al., 2004; Leitze et al., 2005). It is even hypothesized to possibly aggravate the development of osteoarthritis, however, no positive correlation has been found yet (Conteduca et al., 1991; Jonsson et al., 2004; Leitze et al., 2005).

Persevering ALRI may be a result of injury to the anterolateral structures of the knee. Therefore, an additional lateral extra-articular tenodesis (LET) or a reconstruction of the anterolateral ligament (ALL) is proposed by multiple authors to overcome the problem of rotational instability (Hewison et al., 2015; Slette et al., 2016; Sonnery-Cottet et al., 2017; Sonnery-Cottet et al., 2015). However, no reliable diagnostic tool is available for identifying patients with ALRI of the knee, while such a tool is essential in order to perform a reliable diagnosis and evaluation of the possible effectiveness of such treatments.

At this moment, the main clinical tests to diagnose ALRI are the pivot shift test (Galway & MacIntosh, 1980) and anterior drawer test with the foot in 30° of internal rotation (Larson, 1983; Slocum & Larson, 1968). Other tests, such as Slocum’s test (Slocum et al., 1976), Losee test (Losee et al., 1978) and jerk test (Hughston et al., 1976a) are comparable to the pivot shift test. These tests mainly demonstrate anterior subluxation of the lateral tibia plateau on the lateral femoral condyle. The pivot shift test is an accurate diagnostic test for rupture of the ACL with a sensitivity ranging from 0% to 93% and specificity ranging from 82% to 100% (Benjaminse et al., 2006; Leblanc et al., 2015). Even though the pivot shift test assesses rotatory laxity of the knee additional to anteroposterior laxity, no distinction can be made between the amount of anteroposterior or rotatory laxity. To which extent the pivot shift contributes in specifically diagnosing ALRI as a consequence of injury to secondary constraints is unclear (Bonanzinga et al., 2017).

Although there is no reliable clinical test for assessment of ALRI of the knee, multiple ex vivo studies have shown that passive internal rotation of the knee is increased at flexion angles greater than 30° if anterolateral structures, in particular the ALL, are severed in the ACL-deficient knee (Bonanzinga et al., 2017; Dodds et al., 2014; Kittl et al., 2015; Monaco et al., 2012; Parsons et al., 2015; Sonnery-Cottet et al., 2016; Wroble et al., 1993). Passive internal rotation tests of the knee have only been performed in subjects while trying to validate new tools for assessing rotation of the knee (Colombet et al., 2012; Mouton et al., 2012). However, these are mostly expensive tools, time consuming and not easily applicable.

Therefore, we think that ALRI of the knee might also be demonstrated during physical examination with a tibial internal rotation test (TIR test). The purpose of this study is to determine the practicability and rater agreement reliability of a tibial internal rotation test for assessment of internal rotatory instability as part of ALRI in patients with a distorsion of the knee, suspected for ACL injury, in whom rotatory instability should be assessed.

## Methods

Between May and November 2016 a monocenter study was performed for evaluating rater agreement reliability of the TIR test. Inclusion criteria were patients between 18 and 50 years of age with a history of distorsion of the knee and thus strong suspicion on ACL injury or patients with proven ACL injury on magnetic resonance imaging (MRI). Patients with a history of previous injury of the knee or lower limb, a locked knee, complaints of the contralateral knee, rheumatoid disease or other inflammatory disease of the joints, congenital lower limb malformation that could influence rotation of the leg or foot, asymmetrical rotation of the hips and asymmetrical leg axes were excluded. All subjects provided written informed consent and the study was approved by the institutional review board of the author’s institution.

### Procedure

Two examiners independently performed physical examination of both knees of each patient to assess internal rotation of the lower legs. The first examiner was one of three experienced orthopaedic surgeons participating in the study. The other examiner was a well-trained medical student blinded for the affected knee. Prior to assessment of the knee, each patient was assessed for symmetric hip rotation and long leg axis.

### Physical examination of the knee

Physical examination of the knee consisted of the tibial internal rotation test as described below. Furthermore, the dial test in prone position (Veltri & Warren, 1994), the pivot shift test performed according to Galway and MacIntosh (Galway & MacIntosh, 1980), anterior drawer test with foot in 30° internal rotation, neutral position and 15° external rotation (Larson, 1983; Slocum & Larson, 1968) and Lachmann test (Rossi et al., 2011) were performed. The knees were also assessed for effusion, range of motion, collateral instability (Hughston et al., 1976a) and meniscal injury (Rossi et al., 2011). All tests were scored according to the International Knee Document Committee (IKDC) criteria (Hefti et al., 1993). To objectify true rotation of the knees, internal and external rotation tests were also performed using a quantitative measuring device. All tests were performed in consecutive order to prevent unblinding of the blinded examiner. Physical examination of patients was repeated during a second appointment for assessing the intra-rater agreement. The examiner was blinded for the outcomes of the previous physical examination.

### Tibial internal rotation test (TIR test) of the knee

**Fig. 1:**
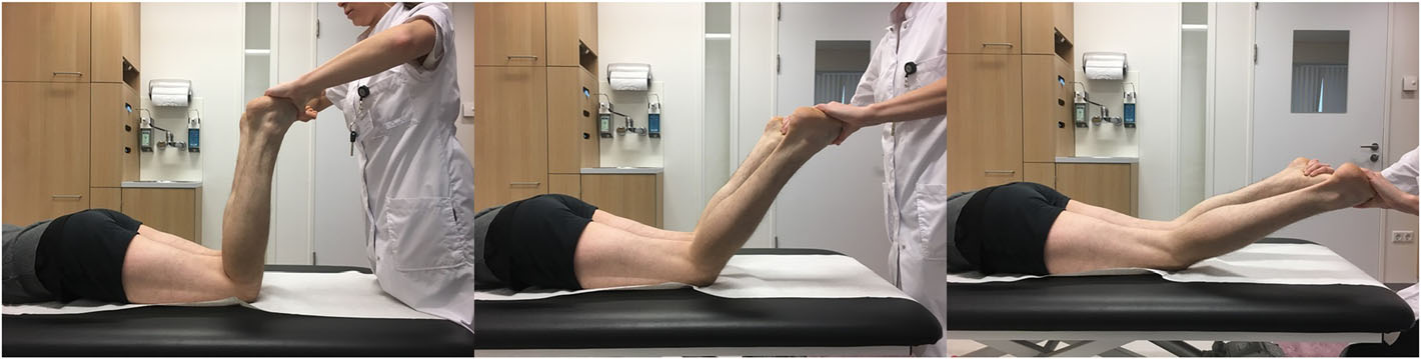
Performing the TIR test in 90°, 60° and 30° of flexion of the knees

**Fig. 2:**
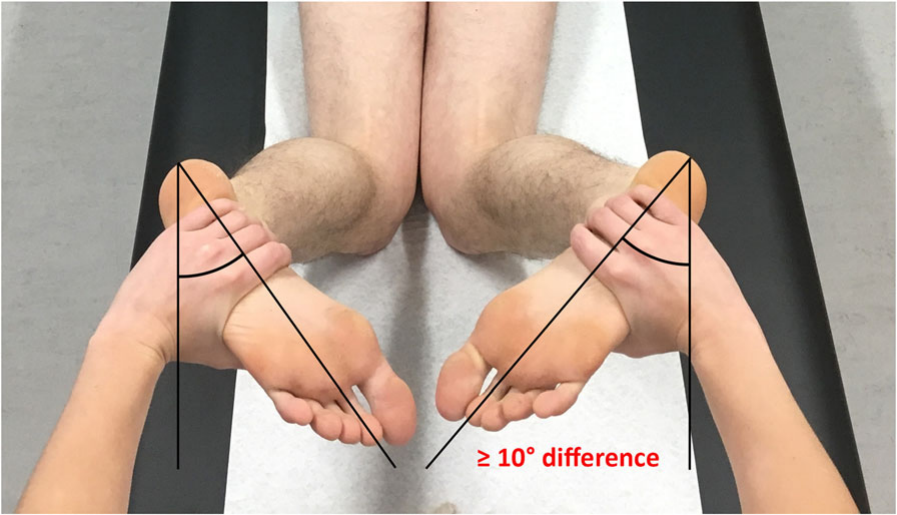
Positive TIR test of the right knee in 60° of flexion of the knees


The TIR test of the knee was performed in prone position. This position allows easy adaptations of the knee flexion angle, good control of the hip extension angle (Mouton et al., 2012) and requires only one examiner. The internal rotation of the lower leg was tested in 30°, 60° and 90° of flexion of the knees (Fig. 1). The flexion angles were based on data from biomechanical studies (Bonanzinga et al., 2017; Dodds et al., 2014; Kittl et al., 2015; Monaco et al., 2012; Parsons et al., 2015; Sonnery-Cottet et al., 2016; Wroble et al., 1993; Zantop et al., 2007). Both knees were positioned directly next to each other and the hips were slightly internally rotated. Internal rotation torque was manually applied on both feet by the examiner, holding the heel. Care was taken to have the ankle in a neutral position. The thigh-foot angle was determined with the feet functioning as pointers of tibial internal rotation relative to the femur (Fig. 2) (Loomer, 1991). Slight internal rotation of the hip was necessary to prevent the feet from touching each other whilst internally rotating the tibia. Based on the dial test (Veltri & Warren, 1994) and biomechanical studies (Sonnery-Cottet et al., 2016; Tsai et al., 2008; Wroble et al., 1993), a ≥10° side-to-side difference in thigh-foot angle was considered a positive TIR test (Fig. 2). An additional video file shows this in more detail (see Additional file 1). The test was either scored positive or negative. Attempting to control manual torque equally on both sides the patients were asked whether torque was applied equally on the left and right knee.

**Additional file 1:** Performing the TIR test in 60° of flexion of the knees, with a positive test on the right knee. (MOV 11468 kb)


### Quantification of knee rotation

**Fig. 3:**
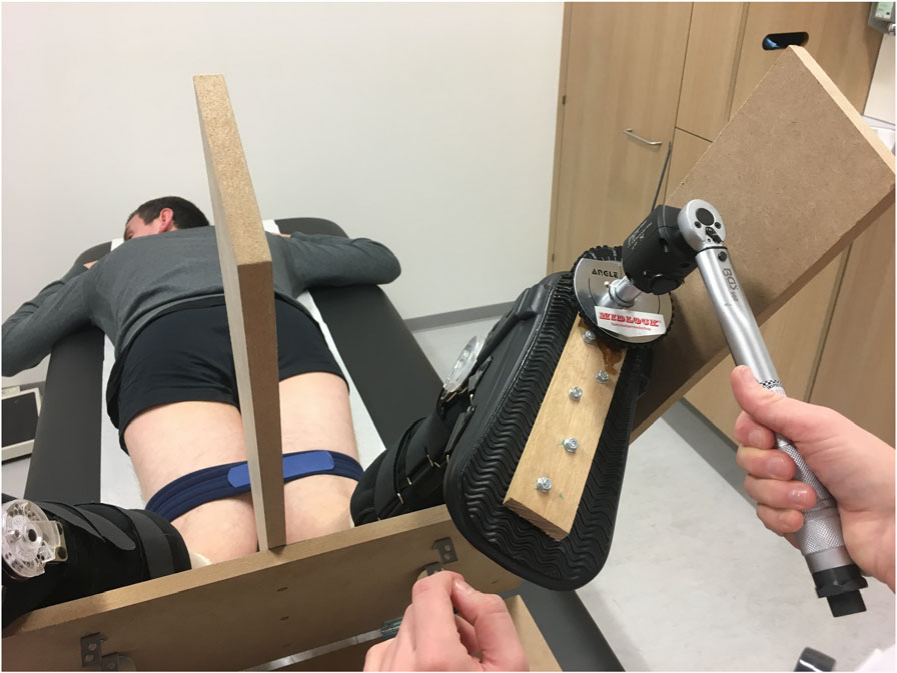
Internal rotation with 6 Nm torque

For quantification of the amount of internal rotation a device was developed (Fig. 3) (Colombet et al., 2012; Mouton et al., 2012). The patient was placed in prone position with the knees in 30°, 60° and 90° of flexion. Support was provided for the legs while keeping accurate flexion angles of the knees and a fixed amount of internal rotation of the hips necessary for internal rotation of the tibia such that relative muscle relaxation was possible. Air inflatable walkers (protect.Air ROM Walker, size medium, Medi, Bayreuth, Germany) were used to fixate the ankle relative to the tibia to minimize the rotation of the ankle. It offers a snug fit without being uncomfortable. A strap was used on both legs just proximal to the knees to avoid natural abduction of the hips. Based on biomechanical studies in vivo*,* a 6 Nm external rotation and internal rotation torque was applied on both feet by means of a wrench with an electronic torque adapter (Kraftwerk Europe, art. 4081–14, ±2%) (Alam & Bull, 2013; Branch et al., 2010; Markolf et al., 1984; Mouton et al., 2012; Shultz et al., 2007; Tsai et al., 2008). With help of fixed angle gauges the thigh-foot angle was measured during internal and external rotation of the ACL-insufficient knee and contralateral knee with both feet starting in neutral position and not the patient’s own resting position of the feet. A side-to-side difference of ≥10° after applying 6 Nm of internal torque was considered a positive TIR test on the device. Also full range of rotation was calculated.

### MRI of the ALL and anterolateral structures

The ALL was retrospectively identified on MRI. The ALL was scored as visualized or non-visualized. If the ALL was visible, it was scored intact or severed including the location of the lesion (proximal or distal of the lateral meniscus). Also, the anterolateral capsule, lateral collateral ligament (LCL), ALL and iliotibial band (ITB) were assessed for surrounding edema, indicating possible injury to the anterolateral structures of the knee. In addition, the MRI was also assessed for other injury of the knee by a musculoskeletal radiologist as part of routine protocol in patients with ACL injury.

### Statistical analysis

Statistical analysis was performed using Statistical Package of the Social Sciences (SPSS, Chicago, IL, Version 24.0). Because of absence of a gold standard to assess for accuracy of the TIR test, we focused on the degree of agreement between different raters. Primary outcomes are intra-rater agreement, inter-rater agreement and rater-device agreement of the TIR test, secondary outcomes are correlations between the pivot shift test, the anterior drawer test with foot in internal rotation and the TIR test. To determine consistency among raters Cohen’s kappa coefficients (κ_C_) were calculated for intra-rater agreement and rater-device agreement (Cohen, 1960). For assessing intra-rater agreement of the pivot shift test a weighted kappa coefficient (κ_w_) was calculated (Cohen, 1968). For determining inter-rater agreement the two raters are considered not unique. Rater number one was considered one of three participating orthopaedic surgeons, therefore Fleiss’ kappa coefficients (κ_F_) were calculated (Fleiss, 1971). For comparing results of the pivot shift test versus the tibial internal rotation test a rank biserial correlation coefficient was calculated. For comparing a grade II-III pivot shift and positive anterior drawer test with foot in internal rotation to the TIR test the Fisher’s exact test was used.

The kappa values are typically interpreted as follows: <0.00, poor agreement; 0.00–0.20, slight agreement; 0.21–0.40, fair agreement; 0.41–0.60, moderate agreement; 0.61–0.80, substantial agreement; and 0.81–1.00, almost perfect agreement (Landis & Koch, 1977).

The prevalence of a positive TIR test in patients with ACL injury is unknown. Therefore, no sample size was calculated. Our aim was to include a minimum of 50 subjects (Donner, 1998). Literature on sample size estimation techniques is limited for rater agreement (Donner, 1998; Donner & Eliasziw, 1992; Rotondi & Donner, 2012).

## Results

**Fig. 4:**
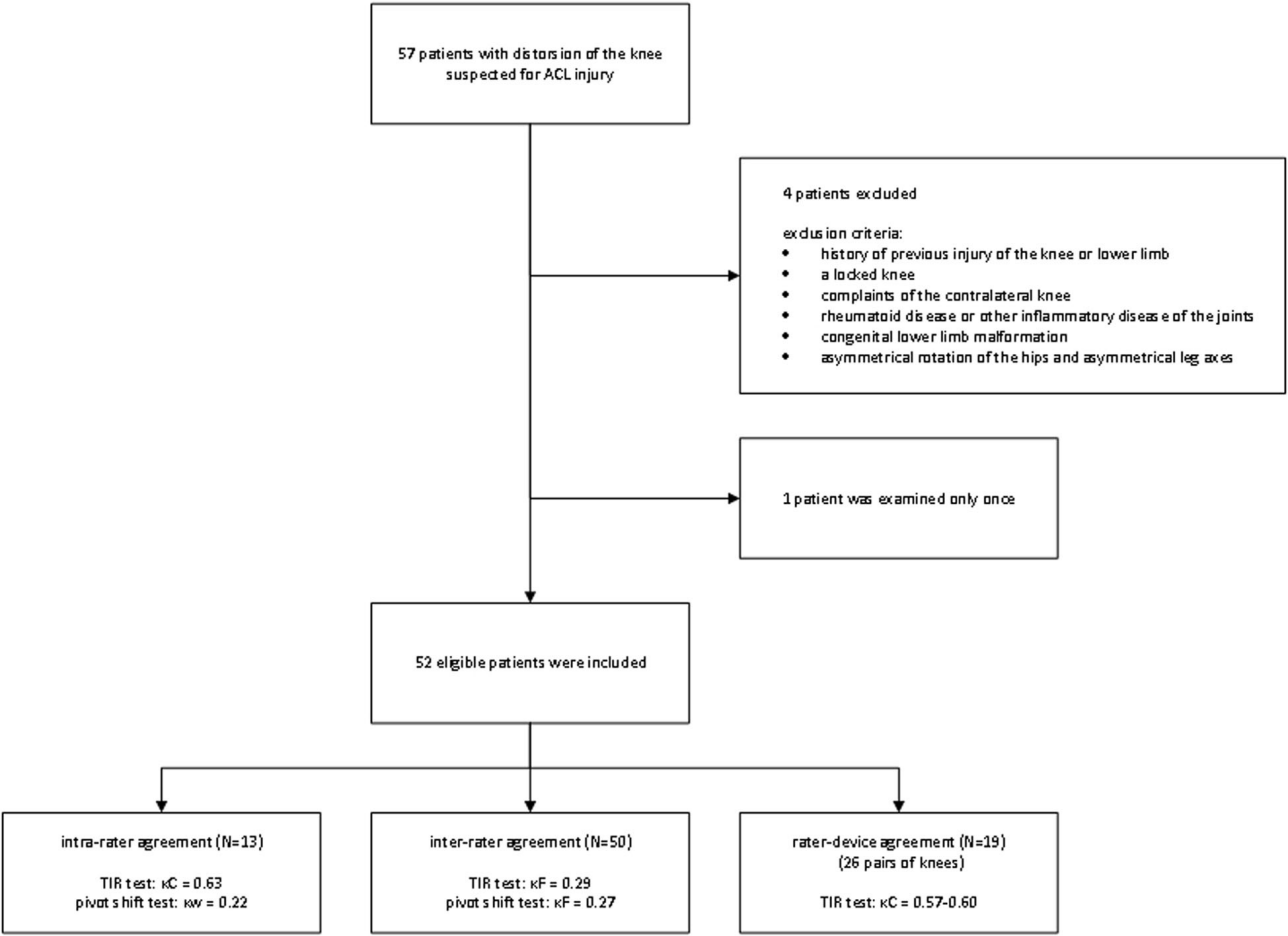
Flowchart of patient selection for rater agreement reliability of the TIR test and the pivot shift test

In this study, 57 patients were included, however, after physical examination 4 patients turned out to meet one of the exclusion criteria and were therefore excluded from the study. Another patient was examined only once by the researcher and was also excluded. Fifty (96%) patients were assessed for inter-rater agreement and 13 (25%) patients for intra-rater agreement. For rater-device agreement 19 (37%) patients were assessed of whom 7 were assessed again during second physical examination, therefore 26 pairs of knees were used (Fig. 4). In 7 (13%) patients the examiner was not blinded, because the patient accidentally gave out the affected side or was using crutches or an orthosis. For patients characteristics see Table 1.

**Table 1:** Patient characteristics of 52 patients

gender (men/women)	31 (59.6%)/21 (40.4%)
mean age in years ± SD	29.9 ± 9.2
mean body mass index in kg/m^2^ ± SD	24.4 ± 2.9
mean time between trauma and physical examination in weeks ± SD	23.4 ± 31.6
≤6 weeks	12 (23.1%)
>6 weeks	39 (75%)
no recollection of trauma	1 (1.9%)
mean time between first physical examination and second physical examination in weeks ± SD	5.6 ± 3.8
mean time between trauma and MRI in weeks ± SD	16.4 ± 26.1
injured side (left/right)	20 (38.5%)/32 (61.5%)
unblinding of researcher	7 (13.5%)
inability of relative muscle relaxation	19 (36.5%)
physical therapy prior to first physical examination	37 (71.2%)
treatment	
ACL reconstruction	29 (55.8%)
additional lateral extraarticular tenodesis^a^	4 (13.8%)
conservative treatment	18 (34.6%)
diagnostic trajectory	5 (9.6%)

^a^Lemaire or modified Lemaire procedure

All included patients showed increased anterior translation as assessed by the Lachmann test, anterior drawer test or pivot shift test when performed by either the orthopaedic surgeon or blinded examiner. No increased posterior translation was found as assessed by the posterior drawer test.

Mean internal rotation, external rotation and total rotation are presented in Table 2. The maximum mean internal rotation was 32.2° in 30° of knee flexion. No statistically significant rotational differences were found between injured and healthy knee.

**Table 2:** The amount of tibial rotation in degrees in 26 pair of knees suspected for unilateral ACL injury with a 6 Nm torque

	injured side	intact side	mean difference^a^
30°of flexion			
internal rotation	32.2 ± 11.8	32.2 ± 9.7	0.0 ± 11.1
external rotation	39.4 ± 9.6	37.3 ± 9.6	2.1 ± 8.4
total rotation	71.6 ± 17.3	69.4 ± 16.5	2.2 ± 15.0
60° degrees of flexion			
internal rotation	24.5 ± 7.8	25.5 ± 7.5	−1.1 ± 8.5
external rotation	39.7 ± 6.7	38.0 ± 8.9	1.8 ± 8.5
total rotation	64.2 ± 11.8	63.5 ± 13.8	0.7 ± 14.0
90° degrees of flexion			
internal rotation	24.1 ± 8.9	25.3 ± 9.4	−1.2 ± 12.3
external rotation	40.8 ± 8.1	39.1 ± 10.2	1.7 ± 7.2
total rotation	64.9 ± 11.6	64.4 ± 15.3	0.5 ± 13.8

^a^Not statistically significant

The TIR test was easily applicable by the examiner and the patient did not experience any discomfort during the test.

In total the blinded examiner found a positive TIR test on the injured knee in 10 (19.2%) patients and found a positive test on the healthy knee in 7 (13.5%) patients during the first physical examination. The orthopaedic surgeon found a positive TIR test in 5 (9.5%) patients, all in the injured knee. The overall proportion of agreement between raters was 82%. On the internal rotation device 6 (23.1%) of 26 pair of knees had a positive test on the injured knee. Overall agreement between the blinded examiner and the device was 65%.

Fair inter-rater agreement was found for the TIR test in 30°, 60° and all flexion angles combined, κ_F_ = 0.29 (95% CI: 0.02 to 0.57), *p* = 0.04 (Table 3).

Substantial intra-rater agreement was found for 30°, 60°, and all flexion angles combined, κ_C_ = 0.63 (95% CI: -0.02 to 1.28), *p* = 0.02. No kappa could be calculated for 90° of flexion, because no positive TIR test was found during the second examination.

A moderate to substantial rater-device agreement was found for 30°, 60°, 90° and all flexion angles combined by calculating Cohen’s kappa. Their respective values are κ_C_ = 0.60 (95% CI: -0.19 to 1.01), *p* = 0.002 for 30° and 60° of flexion in the knees. For 90° of flexion κ_C_ = 0.62 (95% CI: 0.15 to 1.10), *p* = 0.001. For all flexion angles combined κ_C_ = 0.57 (95% CI: 0.20 to 0.94), *p* = 0.003.

**Table 3:** Rater-agreement of the TIR test and the pivot shift test

	N=	κ	SE	95% confidence interval	*P* value
intra-rater (κ_C_)	13				
30° of flexion		0.63	0.33	− 0.02-1.28	0.015
60° of flexion		0.63	0.33	−0.02-1.28	0.015
90° of flexion^a^		–			
all flexion angles combined		0.63	0.33	−0.02-1.28	0.015
pivot shift test (κ_w_)		0.22	0.39	−0.55-0.99	n.s.
inter-rater (κ_F_)	50				
30° of flexion		0.29	0.14	0.02–0.57	0.038
60° of flexion		0.29	0.14	0.02–0.57	0.038
90° of flexion		−0.08	0.14	−0.35-0.20	n.s.
all flexion angles combined		0.29	0.14	0.02–0.57	0.038
pivot shift test		0.27	0.09	0.09–0.44	0.003
rater-device (κ_C_)	26				
30° of flexion		0.60	0.21	0.19–1.01	0.002
60° of flexion		0.60	0.21	0.19–1.01	0.002
90° of flexion		0.62	0.24	0.15–1.10	0.001
all flexion angles combined		0.57	0.19	0.20–0.94	0.003

^a^No kappa coefficient could be calculated

*n.s.* not significant

For physical examination tests in relation to a positive and negative TIR test see Table 4. From 11 patients with a positive TIR test in 30°, 60°, and/or 90°, 2 (18.2%) patients had an additional MCL lesion. From 41 patients with a negative TIR test, 17 (41.5%) patients had additional injury to one of the collateral ligaments.

**Table 4:** Physical examination in 52 patients suspected for ACL injury with a positive and negative TIR test

	positive TIR test^a^ (*N* = 11)	negative TIR test^a^ (*N* = 41)
pivot shift test^a,b^		
0 to 1+	–	4 (9.8%)
1+ to 2+	6 (54.5%)	19 (46.3%)
2+ to 3+	3 (27.3%)	10 (24.4%)
3+	–	1 (2.4%)
no assessment possible	2 (18.2%)	7 (17.1%)
anterior drawer test with foot in internal rotation^a^		
negative	4 (36.3%)	26 (63.4%)
positive	7 (63.6%)	15 (36.6%)
varus gapping^a,c^		
grade A	11 (100.0%)	34 (82.9%)
grade B	–	5 (12.2%)
grade C	–	2 (4.9%)
grade D	–	–
valgus gapping^a,c^		
grade A	9 (81.8%)	31 (75.6%)
grade B	2 (18.2%)	9 (22.0%)
grade C	–	1 (2.4%)
grade D	–	–

^a^Assessed by either the blinded examiner and/or orthopaedic surgeon

^b^0 (normal; negative), 1+ (nearly normal; glide), 2+ (abnormal; clunk), 3+ (severely abnormal; gross), according to the 2000 IKDC objective knee examination score

^c^Grade A (normal; 0–2 mm), grade B (nearly normal; 3–5 mm), grade C (abnormal; 6–10 mm), grade D (severely abnormal; > 10 mm), according to the 2000 IKDC objective knee examination score

There is no statistically significant association between a pivot shift grade I-III and grade II-III and a positive TIR test in 30°, 60°, 90° of knee flexion and all flexion angles combined as assessed by the Fisher’s exact test. Also no statistically significant association was found between a positive TIR test and a positive anterior drawer test with foot in 30° of internal rotation. A rank biserial correlation did not show a statistically significant correlation between the pivot shift test and the TIR test for all flexion angles of the knee and all flexion angles combined (Table 5).

**Table 5:** Correlation between the TIR test and other diagnostic tests for ALRI

	pivot shift test (*N* = 52)		pivot shift test grade II-III (*N* = 33)	anterior drawer test with foot in internal rotation (N = 52)
	r (rb)	*P* value	FE (*P value)*	FE (*P value)*
30° of flexion	0.05	0.815	1	0.264
60° of flexion	0.05	0.815	1	0.264
90° of flexion	0.16	0.503	1	0.379
all flexion angles combined	0.05	0.815	1	0.264

r(rb) = rank biserial correlation

FE = Fisher’s exact test

Based on the pivot shift test grade II and III, 13 (34%) patients were diagnosed with ALRI by the orthopaedic surgeon compared to 21 (64%) patients diagnosed by the blinded examiner. The overall proportion of agreement between examiner and orthopaedic surgeon was 48%. The pivot shift test showed similar inter-rater agreement compared to the tibial internal rotation test with κ_F_ = 0.27 (95% CI: 0.09 to 0.44), *p* = 0.003 (Table 3).

Of 50 (96%) patients the MRI of the ACL-injured knee was retrospectively reviewed, with mean time between trauma and MRI being 16.4 ± 26.1 weeks. From two patients the MRI was not assessable. Of all patients, 44 (88.0%) patients had ACL injury on MRI. In 36 (81.8%) patients the ALL was visualized on MRI. Because of poor imaging, 6 MRIs were not suitable for assessment of the ALL. In 7 (19.4%) cases the status of the ALL was rated as abnormal of which 4 (57%) had a distal lesion. In 26 (52.0%) patients slight edema surrounding at least one of the anterolateral structures, such as the anterolateral capsule, LCL, ALL or ITB, was found. For concomitant injury seen on MRI, see Table 6.

**Table 6:** Features on MRI of the injured knee of 50 patients

ACL	
intact	4 (8%)
contusion	2 (4%)
partial rupture	9 (18%)
rupture	35 (70%)
PCL	
intact	46 (92%)
contusion	1 (2%)
buckling	3 (6%)
LCL	
intact	44 (88%)
sprain	5 (10%)
partial rupture	1 (2%)
MCL	
intact	38 (76%)
sprain	7 (14%)
partial rupture	1 (2%)
rupture	4 (8%)
lesion of the lateral meniscus	13 (26%)
lesion of the medial meniscus	20 (40%)
ALL	
visualized	36 (72%)
normal	29 (80.6%)
abnormal	7 (19.4%)
non visualized	8 (16%)
no assessment possible	6 (12%)
edema surrounding anterolateral structures^a^	26 (52%)

^a^Slight edema surrounding at least one of the following structures: anterolateral capsule, LCL, ALL and/or ITB

## Discussion

This study is the first to investigate manual application of internal torque to the tibia as a physical diagnostic test for ALRI in a representative group with high incidence of ACL rupture. The TIR test is easily applicable and no discomfort is experienced by the patient.

Fair inter-rater agreement, substantial intra-rater agreement and a moderate to substantial rater-device agreement for the TIR test were found. No significant correlations were found between the TIR test and the pivot shift test and anterior drawer test with foot in internal rotation. This strengthens the idea that the TIR test assesses a different aspect of ALRI than the aforementioned tests do. The TIR test only demonstrates increased tibial internal rotatory laxity and can therefore be of additional value to the dynamic pivot shift test.

Some inaccuracies of the TIR test might occur. When manual torque is applied to the foot, rotation and supination occurs in the ankle joint. However, assuming that there are no side-to-side differences in ankle joint motion, this should have no effect on the outcome of a positive or negative TIR test. Also, lower limb malformation that could influence rotation of the leg or foot was an exclusion criterium. In addition, it is possible that the position of the ankle could have influenced the rater-device agreement, since positioning can differ between the manual TIR test and internal rotation performed on the device and patients were more able to relax while perfoming internal rotation on the device. We have chosen a cut-off value of ≥10° side-to-side difference based on the dial test and in our opinion a ≥10° side-to-side difference can be estimated without help of an additional goniometer. This value can be influenced by individual variety of rotation laxity and applied torque of the examiner. We do not know if this results in overestimation or underestimation of positive results of the TIR test.

Other inaccuracies may occur due to the patient’s reflex resisting instability tests due to discomfort. Also, secondary stabilizers contributing to the function of the primary stabilizers might decrease the magnitude of the instability demonstrated by the clinical test (Larson, 1983; Noyes et al., 1980). Greater knee laxity is thought to be associated with an increased demand of leg musculature to maintain joint stability (Shultz et al., 2007). Therefore, this effect can probably be even more attributed to patients that have had physical therapy.

In 7 (13.5%) patients examined by the blinded examiner a positive TIR test was found on the contralateral healthy knee. These outcomes were later scored as a negative test for determining inter-rater agreement, because during this study and in a clinical setting the orthopaedic surgeon is never blinded for the affected side. Also some patients notified after examination that they had unwittingly protected their affected knee. Other biasing factors of static and dynamic rotatory laxity are time interval between trauma and the physical examination and the time interval for assessing intra-rater agreement reliability. Both these factors lead to differences in the amount of effusion and pain the patient is experiencing.

Noyes et al. (Noyes et al., 1991) found that the pivot shift test varied substantially between examiners in cadaveric knees. As far as we know, only one study of Labbe et al. (Labbe et al., 2011) reports a kappa value for the inter-rater agreement of the pivot shift test with κ = 0.83. Nonetheless, in their study three clinicians evaluated only 12 subjects of whom four subjects had ACL-intact knees and eight subjects had varying degrees of joint instability due to a rupture of the ACL. This limits the inter-rater agreement to only eight subjects with ACL injury. Also the three clinicians were in total agreement of only five of the 12 subjects (42%) (Labbe et al., 2011). According to our results the pivot shift test has slight inter-rater agreement with a Fleiss’ kappa of 0.27 and an overall agreement of 48%. These dissimilarities in outcomes might be the result of the different sample sizes used, different forces applied by the examiner during the maneuver, and grading of the pivot shift test relies on the examiner’s perception of the change of motion that occurs. This makes it a highly subjective test and patient guarding may limit the reliability of the test (Lane et al., 2008; Noyes et al., 1991). Remarkably, several patients guarded the pivot shift test performed by the blinded examiner, which might be due to experienced discomfort during the maneuver performed by the orthopaedic surgeon beforehand. The subjectivity of the pivot shift test confirms the need for other diagnostic tests for assessing rotatory laxity such as the TIR test.

Comparing our data of internal, external and total rotation to other studies, similar results were found. Mayr et al. (Mayr et al., 2011) found no significant difference in internal and external rotation between ACL-injured and healthy knee. They report on a side-to-side difference in healthy knees of 5.5° and 9.0° of internal and external rotation respectively, while other studies (Alam & Bull, 2013; Mouton et al., 2012) state that physiologic side-to-side differences in tibial rotation averaged between 1.53° and 3.5°. Our study found non-significant side-to-side differences ranging from − 1.2° to 2.2° between healthy and injured knee. Only Markolf et al. (Markolf et al., 1984) report on a statistical significant difference of 3.0° between ACL-deficient and healthy knee concerning the total rotation. Comparing the data remains challenging because of highly variable conditions and testing methodologies (Colombet et al., 2012; Mouton et al., 2012).

Many structures are described that contribute to ALRI and increased internal rotation. Cadaveric biomechanical studies show that the contribution of the ACL in restraining tibial internal rotation torque decreases as the flexion angle of the knee increases while the contribution of the ALL increases, especially in flexion angles ≥30° of the knee (Bonanzinga et al., 2017; Monaco et al., 2012; Parsons et al., 2015; Sonnery-Cottet et al., 2016; Wroble et al., 1993). This suggests that increased passive internal rotation as assessed by the TIR test may be the result of injury of the ALL leading to ALRI. Other structures that also contribute to internal rotatory stability of the knee are the ITB (Kittl et al., 2015; Sonnery-Cottet et al., 2016), the LCL (Parsons et al., 2015; Wroble et al., 1993; Zantop et al., 2007), the medial collateral ligament (MCL), posterolateral structures (Wroble et al., 1993; Zantop et al., 2007) and the menisci (Musahl et al., 2010). The TIR test cannot distinguish between internal rotatory laxity as a result of damage to anterolateral structures or damage to other secondary internal rotatory stabilizers. This has to be taken into account when considering a surgical treatment for adjustment of internal rotatory instability. Therefore, the clinician should interpret the outcome of the TIR test in combination with varus- and valgus gapping. Also, attention must be paid to the movement of the lateral and medial tibial plateau when performing the anterolateral drawer test and posteromedial drawer test in order to distinct increased internal rotatory laxity as a consequence of either anterolateral- or posteromedial rotatory instability (PMRI) (Larson, 1983; Slocum & Larson, 1968). However, PMRI only occurs in the PCL-deficient knee, since if the tibial plateau moves posteriorly during internal rotation the PCL tightens and locks the joint surfaces together (Hughston et al., 1976a).

How to identify patients that will benefit from additional surgical procedures at time of ACL reconstruction, such as an ALL reconstruction or LET is still subject of debate. A clear definition of ALRI with an objective quantification method of injury would be useful, so that indications for reconstruction of the anterolateral capsule can be defined (Colombet et al., 2012; Laprade, 2016). At the moment, the indication for an additional procedure is primarily based on the orthopaedic surgeon’s intuition and experience, while bearing in mind the patient characteristics such as age, laxity and level of sport (Sonnery-Cottet et al., 2017). Also multiple orthopaedic surgeons (Monaco et al., 2012; Sonnery-Cottet et al., 2017) believe that anterolateral structures must be repaired during time of ACL reconstruction if a pivot shift grade ‘2+’ or ‘3+’ is present, even though multiple structures in the knee other than the ALL can influence the highly subjective pivot shift test (Hughston et al., 1976b; Lane et al., 2008). This subjectivity of the pivot shift test can result in a high variety in the decision of whether or not to perform an additional LET or ALL reconstruction amongst orthopaedic surgeons. Since the pivot shift test is also influenced by other structures than the anterolateral, it may be that not all patients identified with ALRI by the pivot shift test may benefit from a LET or ALL reconstruction. Therefore, the TIR test can be a valuable addition in filling up the gap in diagnosing internal rotatory laxity as part of ALRI.

Several studies show that the ALL can be identified on MRI (Caterine et al., 2014; Claes et al., 2014; Devitt et al., 2017; Van Dyck et al., 2016; Helito et al., 2014; Kosy et al., 2015; Porrino et al., 2015; Taneja et al., 2015) and ultrasound imaging (Capo et al., 2017; Cavaignac et al., 2016; Klos et al., 2017; Oshima et al., 2016). These studies show suboptimal results with detection rates on MRI ranging from 51% to 100% in healthy knees and the ACL-deficient knee, with a pooled analysis of 96% (Van Der Watt et al., 2015). Nonetheless, their ability to evaluate injury to the ALL is still unclear and no clinical protocols are yet focusing on assessing the ALL in the ACL-deficient knee. This means that orthopaedic surgeons are still mainly dependable on physical examination. In our study we found similar detection rates of the ALL.

Several limitations are present in this study. First of all, there is no gold standard available for diagnosing ALRI, therefore no sensitivity and specificity could be determined for the TIR test. Also, our measurements could not be compared to a true measurement of rotatory instability of the knee. The consequence is that is not clear if real internal rotatory laxity is assessed by the TIR test. Furthermore, during the examination the orthopaedic surgeon was not blinded. No orthopaedic surgeon had a positive tibial internal rotation test on the healthy contralateral knee in comparison to the blinded examiner. Also, the scoring of laxity by the examiners was in a semi-quantitative fashion and the sensation of laxity and accuracy of grading instability is determined by the examiner’s experience (Larson, 1983). As for the patients, we experienced that not all patients were able to fully relax during physical examination. These aforementioned points may have resulted in false positive or negative testing and consequently in lower kappa values for rater agreement. Also, we do not know whether the time interval between ACL injury and physical examination influences the amount of internal rotation and thus the outcome of the TIR test. No correlation between the ALL and edema of the anterolateral structures on MRI and the TIR test was determined, since there is a lack of a gold standard for identifying the ALL, and heterogeneity of the time intervals between trauma, physical examination and MRI, which could both result in inaccuracies. Also, in the future we would like to compare the results to a healthy control group for further clarification.

Methods of measuring knee kinematics vary greatly and no gold standard exists in assessing ALRI. Further research is needed to develop methods for diagnosing ALRI. Clear definitions of rotatory laxity should be defined before reliable and standardized methods can be developed to quantify dynamic and static rotatory laxity. The focus on quantification devices of tibial rotation seems promising, which can lead to validation of the TIR test in vivo. Also, the ultimate aim is to identify which patients benefit most from additional reconstruction of the anterolateral aspects of the knee and to eliminate concomitant rotatory laxity using an individualized approach for each patient.

## Conclusion

In certain patients with unilateral ACL injury increased tibial internal rotation was shown by means of the TIR test. The TIR test has comparable agreement to the pivot shift test according to our study and can therefore be helpful in demonstrating internal rotatory laxity in addition to the dynamic pivot shift test. A positive TIR test should alert the clinician on possible concomitant injury, however no clinical implications should be based on this test alone. If using the TIR test, we recommend performing the test in prone position with 30°, 60°, and 90° of flexion in the knees in patients with symmetrical position of both feet relative to the tibia. Further research and validation of this novel test is needed.

## Abbreviations

ACL: Anterior cruciate ligament
ALL: Anterolateral ligament
ALRI: Anterolateral rotatory instability
FE: Fisher’s exact
IKDC: International Knee Document Committee
ITB: Iliotibial band
κ_C_: Cohen’s kappa
κ_F_: Fleiss’ kappa
κ_W_: weighted kappa
LCL: Lateral collateral ligament
LET: Lateral extra-articular tenodesis
MCL: Medial collateral ligament
MRI: Magnetic resonance imaging
Nm: Newtonmeter
PMRI: Posteromedial rotatory instability
r(rb): rank biserial correlation
SE: Standard error
SPSS: Statistical Package of the Social Sciences
TIR test: Tibial internal rotation test
